# THE IMPACT OF A PLANT-BASED DIETARY PATTERN ON DEMENTIA RISK: A PROSPECTIVE COHORT STUDY.

**DOI:** 10.1093/geroni/igz038.2691

**Published:** 2019-11-08

**Authors:** Ming-Nan Lin, Tina H Chiu, Chiao-Erh Chang, Ming-Nan Lin

**Affiliations:** 1 Dalin Tzu Chi Hospital, Dalin, Chiayi, Taiwan (Republic of China); 2 Fu Jen Catholic University, New Taipei, New Taipei, Taiwan (Republic of China); 3 National Taiwan University, Taipei, Taipei, Taiwan (Republic of China)

## Abstract

High antioxidants and phytochemicals from plant foods may protect against cognitive decline, while saturated fats from animal based foods considered as risk factors have been associated with dementia. Plant-based diets with limited animal products have been shown to improve glycemic control and reduce diabetes risk. The study aims to examine the association between vegetarian diet and dementia risk in a prospective cohort study. Quantitative data collected from 12,062 participants of Tzu Chi Vegetarian Study from 2005 to 2014 in Taiwan. Data included self-administered questionnaire on demographics, medical history, life-style, and a 57-item food frequency questionnaire (FFQ), National Health Insurance Database and the National Death Registry. Findings show that compared with non-vegetarians, vegetarians had 38% lower risk of dementia (HR = 0.62, 95% CI = 0.39, 0.98) after adjusting for age, sex, education, marital status, physical activities, smoking, alcohol drinking, hypertension, diabetes, hyperlipidemia, and ischemic heart diseases.

